# Genomic Analysis Made It Possible to Identify Gene-Driver Alterations Covering the Time Window between Diagnosis of Neuroblastoma 4S and the Progression to Stage 4

**DOI:** 10.3390/ijms23126513

**Published:** 2022-06-10

**Authors:** Marzia Ognibene, Patrizia De Marco, Stefano Parodi, Mariaclaudia Meli, Andrea Di Cataldo, Federico Zara, Annalisa Pezzolo

**Affiliations:** 1 U.O.C. Genetica Medica, IRCCS Istituto Giannina Gaslini, 16147 Genova, Italy; patriziademarco@gaslini.org (P.D.M.); federicozara@gaslini.org (F.Z.); 2 Scientific Directorate, IRCCS Istituto Giannina Gaslini, 16147 Genova, Italy; stefanoparodi@gaslini.org; 3 U.O.C. Ematologia e Oncologia Pediatrica, Dipartimento di Medicina Clinica e Sperimentale, Università di Catania, 95123 Catania, Italy; mclaudiameli@gmail.com (M.M.); adicata@unict.it (A.D.C.); 4 IRCCS Istituto Giannina Gaslini, 16147 Genova, Italy; annalisapezzolo56@gmail.com

**Keywords:** Neuroblastoma, stage 4S, tumor progression, array-comparative genomic hybridization, whole-exome sequencing, somatic copy number alterations, single-nucleotide variants

## Abstract

Neuroblastoma (NB) is a tumor of the developing sympathetic nervous system. Despite recent advances in understanding the complexity of NB, the mechanisms that determine its regression or progression are still largely unknown. Stage 4S NB is characterized by a favorable course of disease and often by spontaneous regression, while progression to true stage 4 is a very rare event. Here, we focused on genomic analysis of an NB case that progressed from stage 4S to stage 4 with a very poor outcome. Array-comparative genomic hybridization (a-CGH) on tumor-tissue DNA, and whole-exome sequencing (WES) on exosomes DNA derived from plasma collected at the onset and at the tumor progression, pointed out relevant genetic changes that can explain this clinical worsening. The combination of a-CGH and WES data allowed for the identification iof somatic copy number aberrations and single-nucleotide variants in genes known to be responsible for aggressive NB. *KLRB1*, *MAPK3* and *FANCA* genes, which were lost at the time of progression, were studied for their possible role in this event by analyzing in silico the impact of their expression on the outcome of 786 NB patients.

## 1. Introduction

Neuroblastoma (NB) is the most common solid tumor in preschool children [1,2,3]. This embryonal tumor derived from a neural crest shows a great heterogeneity of clinical, biological, genetic, and morphological characteristics [4,5,6]. The International Neuroblastoma Risk Group (INRG) classification system defines the prognosis of NB by various factors at diagnosis: age, histopathological classification, degree of tumor differentiation, amplification of *MYCN* oncogene, DNA ploidy, and presence of segmental chromosome aberrations [7]. Based on the above prognostic factors, NB patients have been classified into five groups: very low-risk, low-risk, intermediate-risk, high-risk, and ultra-high-risk [7,8]. It has been suggested that the tumor microenvironment (TME) might be another independent predictor factor among those currently used to staging and stratifying the treatment of NB patients at diagnosis [9]. TME is a complex network of malignant and nonmalignant cells, which promote NB development, metastasis and resistance to therapy [9]. The tumor-infiltrating lymphocytes have an important prognostic value in NB [10]. NB associated with good prognosis is characterized by a greater number of proliferating T cells and a more structured T-cell organization, which is gradually lost in highly aggressive tumors. Furthermore, it has been shown that highly T-cell-infiltrated tumors are also enriched with both dendritic (DCs) and natural killer (NK) cells, and that their affluence is associated with favorable clinical outcome of NB patients [11]. Notably, it was defined that the expression profiles related to DC and NK cells are strongly associated with survival of NB patients. From the genetic point of view, NB is undoubtedly a genetic disease but its etiopathogenesis is not yet well-known. Many somatic chromosomal imbalances have been highlighted: genomic gains/amplifications or losses as well as whole or segmental chromosome rearrangements [12]. Numerical chromosome alterations (whole chromosome gains or losses) are associated with low-risk disease and favorable outcomes, while segmental chromosome imbalances (partial loss of chromosome arms 1p, 3p, 4p, 6q, 11q, and partial gains of chromosomes arms 1q, 2p, 17q) are associated with high risk of death [13]. There are frequently mutated genes in NB that have not yet been included in the prognostic factors defining the risk of death for the patients [8]. The genes known to be recurrently mutated in NB are: *ALK*, *TERT*, *ATRX*, *LIN28B*, *TP53*, *SMARCA4*, *CDKN1B*, *PHOX2B*, *CHD5*, *MYCN*, *SHANK2*, *PTPRD*, *FGFR1*, *PTPN11*, *NF1*, *NRAS*, *KRAS*, and *BRAF* [6]. Genes coding for the intracellular kinases RAS, RAF, PI3K, MAPK, MEK, AKT, and mTOR are considered among those that mostly influence NB pathogenesis, leading to neoplastic transformation through mechanisms such as apoptosis, proliferation, DNA repair, and angiogenesis [14]. The RAS pathway [15] and the embryonic development-related pathways Wnt, Notch, and Hedgehog pathways [16,17,18] are deeply involved with NB. Note that mutations in RAS/MAPK signaling-pathway genes are described in about 80% of relapsed NB [19].

NB stage 4S defines patients younger than 12 months with localized primary tumors and dissemination limited to specific sites such as liver, skin, and bone marrow (<10% invasion) [20]. This NB stage is characterized by a favorable course of disease and a high rate of spontaneous tumor regression [21,22]. However, very young patients are at risk of rapid progression of liver metastases causing life-threatening compression of lungs, kidneys, inferior vena cava, normal liver tissue, and intestines [23]. The tumor progression to true stage 4 disease is seen occasionally at less than 5 years from diagnosis [24,25].

The ability of NB to differentiate and spontaneously regress may suggest the potential direction for therapeutic intervention. Unfortunately, the mechanisms explaining NB regression or progression are still largely unknown, and it is urgent to identify prognostic markers able to define the behavior of stage 4S tumor.

Here, we studied a child diagnosed as stage 4S NB, who after a 12 months’ tumor regression phase progressed to true stage 4 with **rapid decline in clinical conditions, soon** followed by death. We analyzed by a-CGH the tumor DNA at onset and at progression and, on the basis of our previous findings [26], we applied whole-exome sequencing (WES) technique to DNA extracted from plasma-derived exosomes both at onset and at the time of progression, since exosomal-DNA (exo-DNA) can provide all tumor information. This study allowed us to identify important NB gene-driver mutations at onset that probably triggered the cascade of events leading to the fast worsening of the clinical picture of this patient. Furthermore, we examined some atypical chromosomal losses displayed by tumor DNA at progression, finding two interesting genes, possibly related to NB progression.

## 2. Results

### 2.1. Genomic Profile Analysis

**Figure 1:**
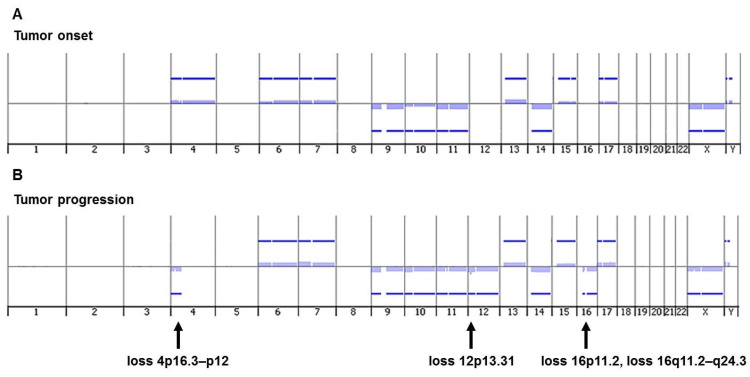
Genomic findings obtained by array-CGH. (**A**) Genomic profile of DNA from tumor biopsy at diagnosis disclosed a numerical genomic profile (NCA) with gain of chromosomes 4, 6, 7, 13, 15, 17, and Y and loss of chromosomes 9, 10, 11, 14, and X; (**B**) Genomic profile of DNA from tumor biopsy at the time of disease progression to true stage 4 highlighted a segmental genomic profile (SCA) with four chromosome losses (loss 4p16.3–p12, loss 12p13.31, loss 16p11.2, loss 16q11.2–q24.3), and 12 numerical whole chromosome abnormalities.

Array-CGH analysis of DNA from primary tumor biopsy at onset showed a numerical genomic profile (NCA) with many whole chromosomes altered, indicating high genomic instability (Table 1 and Figure 1A). Array-CGH analysis of DNA from tumor biopsy at the time of disease progression to true stage 4 disclosed a segmental chromosome profile (SCA). The progressed NB harbored the segmental chromosome alteration typical of NB loss 4p16.3–p12 and the atypical losses 12p13.31, 16p11.2, and 16q11.2–q24.3, with 12 numerical alterations (Table 1 and Figure 1B). The numerical anomalies found in the progressed tumor were substantially the same as those present at onset, except for the presence of the loss of whole chromosome 12, and the lack of gain of whole chromosome 4. Note that chromosome 4 was no longer fully acquired as at the onset of the disease but showed the 4p loss. The a-CGH analysis showed that the tumor genome acquired new segmental chromosomal abnormalities during the disease progression to true stage 4. Notably, the loss 12p13.31 contained the KLRB1 gene [27]; the loss 16p11.2, encompassing the MAPK3 gene, is significantly enriched in NB cases [28]; and the loss 16q contained FANCA [29] and CHD9 [30] genes.

### 2.2. Mutational Profile Analysis

**Figure 2:**
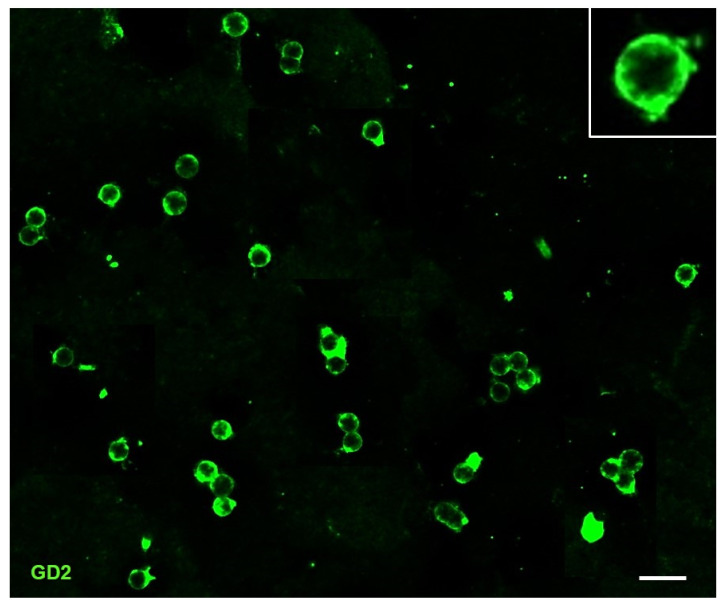
Exosomes derived from peripheral blood samples of the NB patient expressed GD2 marker (green staining). Scale bar: 200 nm. In the inset is visualized an enlarged exosome.

To assess the mutational profile of this rapidly progressed case of NB, WES analysis was performed on DNA from the tumor biopsy at onset and from plasmatic exosomes collected at the time of diagnosis and at the time of disease progression. Recently, our group provided evidence for the presence of double-stranded DNA reflecting the mutational status of parental tumor cells in the exosomes derived from the plasma of the NB patients [26]. We firstly performed an immunofluorescence analysis on the plasma-derived exosomes of the examined patient using the anti-GD2 mAb, a marker specifically expressed on NB cells surface [31], to evaluate their purity. We found that about 95% of NB-derived exosomes expressed GD2 antigen, thus confirming their tumor origin (Figure 2). All NB cells express the GD2 ganglioside; however, this marker is also expressed by CAF-MSC (cancer-associated fibroblast–mesenchymal stromal cells) which are an important source of exosomes [32]. These GD2^+^ exosomes, not derived from NB, did not affect the interpretation of the genetic results as they carry normal DNA, free from both chromosomal and gene mutations [33].

**Figure 3:**
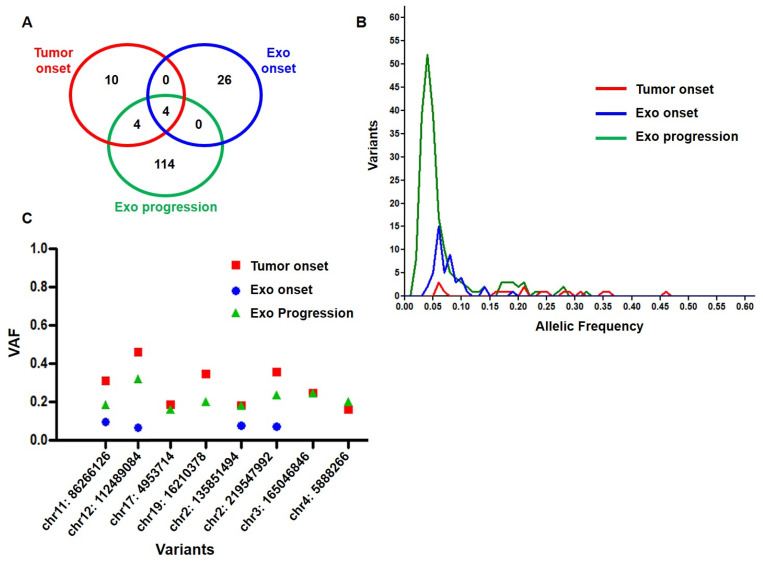
Somatic SNV analysis. (**A**) Venn diagram of somatic SNVs in common among exo-DNA at onset, exo-DNA at progression, and the corresponding tumor DNA at onset. (**B**) Somatic SNV frequency in exo-DNA at onset, exo-DNA at progression, and tumor DNA at onset (**C**) Frequency of the somatic SNVs in common among tumor DNA, exo-DNA at onset and exo-DNA at progression. (VAF = variant allele frequency).

The total somatic single-nucleotide variants (SNVs) we identified on the patient’s exo-DNA were 250, among which 138 were recognized as pathogenic or likely pathogenic variants producing missense, frameshift, stop gained, initiator codon, and splicing mutations, concerning both known NB driver genes and other genes (Supplementary Table S1). A higher overall number of SNVs was identified in the exo-DNA at onset compared to tumor DNA, with most of SNVs found to be exclusive of a single specimen, thus confirming our previous observations [26] (Figure 3A). An impressive increase in SNVs was detected in exo-DNA at progression in comparison with the other analyzed specimens (Supplementary Table S1 and Figure 3A). Furthermore, the exo-DNA at progression had SNVs with higher frequency than the exo-DNA at onset, indicating a clonal evolution inducing the progression itself and the consequent very poor outcome of the patient (Figure 3B). We also evaluated the allelic frequency of the somatic SNVs found in common between the different DNA specimens, with four concordant mutations between tumor DNA and exo-DNA at progression and other four in common among the three specimens (Figure 3C). The WES results therefore confirmed that parental tumor SNVs were detectable in the exo-DNA even with lower frequency.

**Figure 4:**
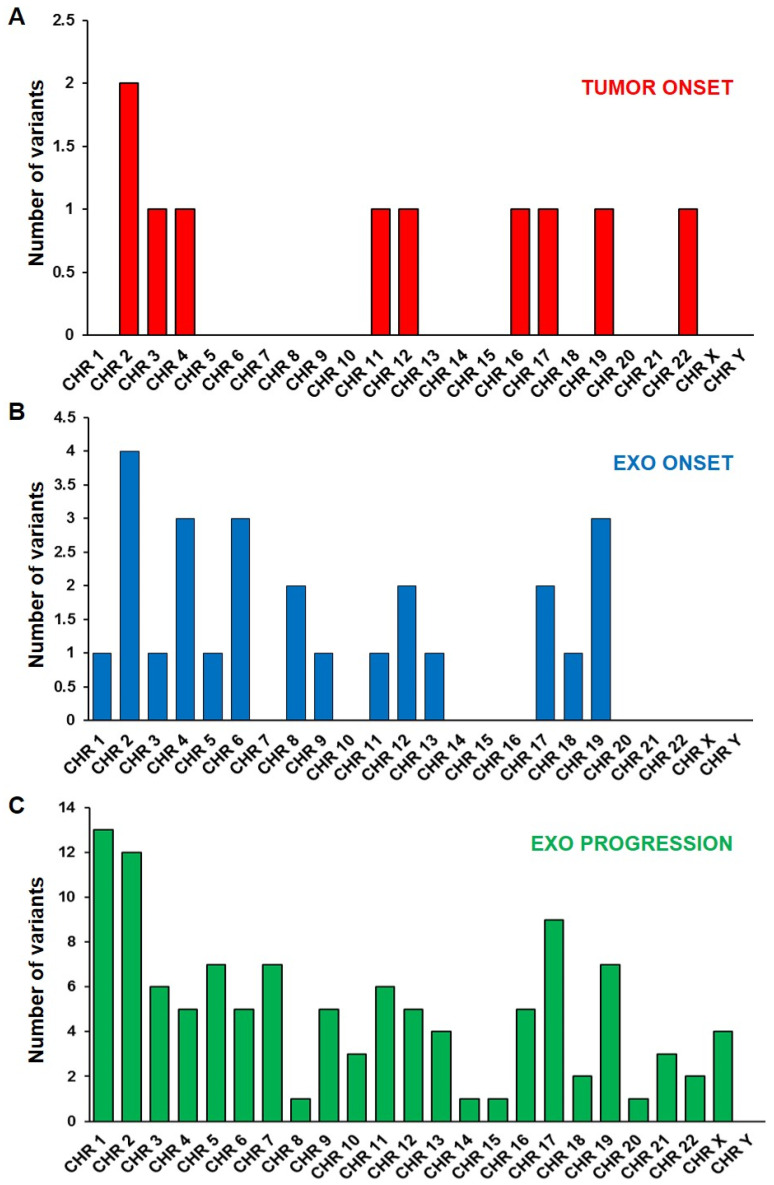
Chromosomes’ localization of somatic SNVs in (**A**) tumor DNA at onset, (**B**) exo-DNA at onset, and (**C**) exo-DNA at disease progression.

**Figure 5:**
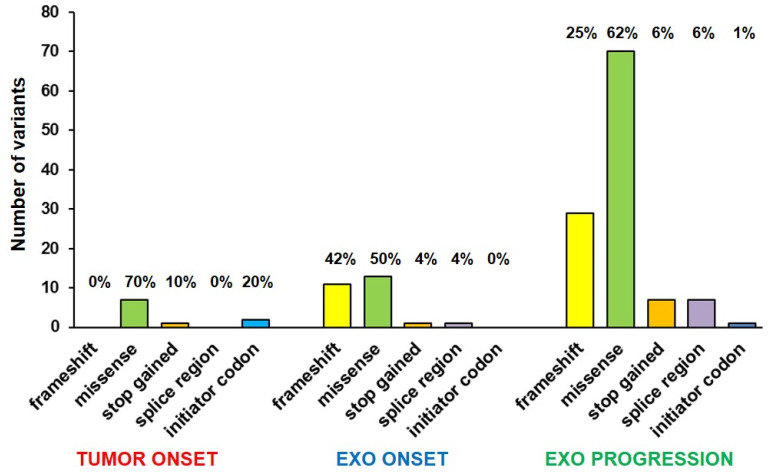
Mutation types in tumor DNA and in exo-DNA. Percentages are calculated on the total number of mutations present in each sample.

WES analysis covered all chromosomes, finding SNVs only in a few chromosomes of tumor DNA, while more chromosomes were involved in exo-DNA at onset. All 24 chromosomes were affected by SNVs in exo-DNA at progression, especially chromosomes 1 and 2 (Figure 4). The highest number of variants was represented in all samples by SNPs causing missense mutations, followed by nucleotide insertions or deletions causing frameshift mutations (Figure 5). In particular, exo-DNA at progression displayed a very large number of missense mutations in comparison to tumor DNA and exo-DNA at onset and with the highest percentage among all kinds of mutations detectable in that single specimen (Supplementary Table S1 and Figure 4). Notably, exo-DNA at progression showed some variants in NB driver genes as *CASZ1*, *PBX2*, *DMBT1*, and *PTPN11* [6], but even more importantly, exo-DNA at onset already displayed pathogenic mutations on the NB driver genes *PTPN11* and *FGFR1.* The *PTPN11* mutation was also present on tumor DNA at onset (Supplementary Table S1).

The somatic CNVs analyzed within the known driver genes related to NB were investigated [6,34,35]. We found that some genes of interest were contained in CNVs identified in the exo-DNA at the time of disease progression to true stage 4. In particular, the *BRAF* gene, involved in the RAS/MAPK pathway, that lies within the 7q duplicated region, the *NF1* gene, that localizes within the 17q duplicated region, and the *ATRX* gene, mapping within the small deletion on chromosome X (Table 2).

### 2.3. Association between 786 NB Patient Survival and Expression of KLRB1, FANCA and MAPK3 Genes

By a-CGH analysis on tumor DNA at progression, we found the loss 12p13.31, containing KLRB1 gene, the loss 16p11.2 containing MAPK3 gene, and the loss 16q11.2–q24.3, containing FANCA gene. We decided to investigate the role of these three genes on NB progression and relapse, since they have all been related to cancer development and progression [27,28,29]. Therefore, we analyzed in silico the impact of their expression on the outcome of 786 NB patients using a large dataset integrated from three different platforms.

#### 2.3.1. Association between Patient Survival and Expression of KLRB1 Gene

**Figure 6:**
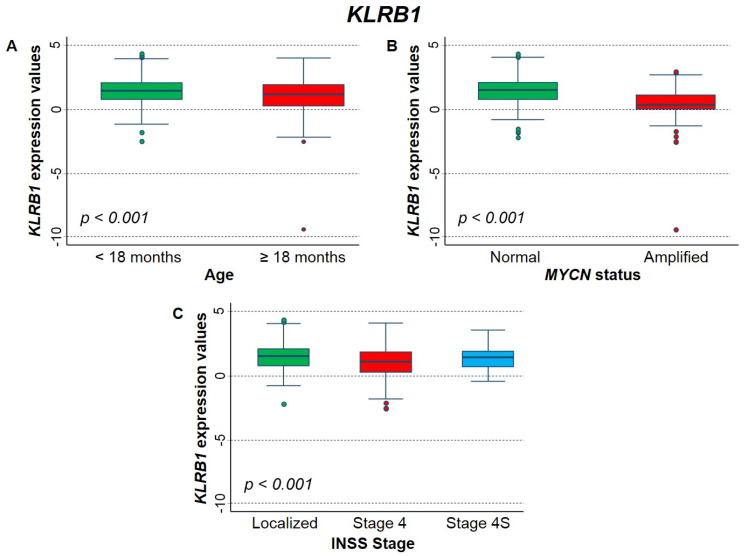
Association between *KLRB1* gene expression and the main patient characteristics at diagnosis evaluated on the entire cohort. (**A**) Age; (**B**) *MYCN* status; (**C**) INSS stage (International Neuroblastoma Staging System).

Figure 6 shows the association between *KLRB1* gene expression and the main patient characteristics at diagnosis. Lower values were significantly associated to higher age (Figure 6A), *MYCN* amplification (Figure 6B), and stage 4 (Figure 6C).

**Figure 7:**
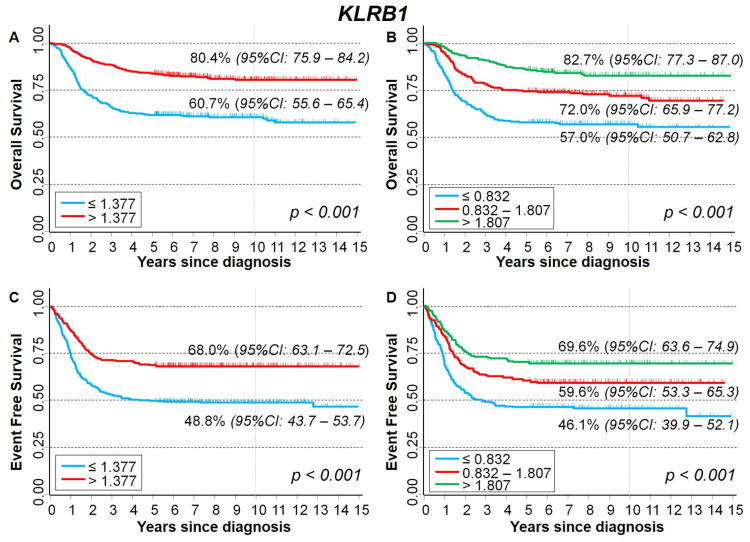
Kaplan–Meier survival curves of the whole cohort of 786 NB patients in relation to *KLRB1* gene-expression values. (**A**) Overall survival, cut-off based on the median expression value; (**B**) Overall survival, cut-offs based on tertile expression values. (**C**) Event-free survival, cut-off based on the median expression value; (**D**) Event-free survival, cut-offs based on tertile expression values. Ten-year survival estimates are displayed.

Figure 7 shows the Kaplan–Meier survival curves of the cohort of NB patients analyzed in relation to *KLRB1* expression levels. Higher values were associated with a better survival for both overall survival (OS) and event-free survival (EFS). In details, OS was 80.4% vs. 60.7% using the median expression value as cut-off (Figure 7A), and 82.7%; and 72.0% and 57.0% after stratification by the tertile values (Figure 7B). The corresponding figures for EFS were 68.0% and 48.8% (cut-off: median value, Figure 7C); and 69.6%, 59.6%, and 46.1% (cut-off: tertile values, Figure 7D). After adjusting for the potential confounding by age, *MYCN* status and stage at diagnosis, the observed associations were confirmed, but statistical significance was borderline for both OS and EFS (Table 3).

The analysis after stratification by *MYCN* status indicates that the association between *KLRB1* expression levels and patient survival was limited to patients with not-amplified *MYCN* (Supplementary Table S2). An apparent protective effect of high gene-expression values was observed both in localized and in stage 4 patients, but the size of sub-cohort of Stage 4S patients was small and very few events were observed in this group (Supplementary Table S3).

#### 2.3.2. Association between Patient Survival and Expression of FANCA Gene

**Figure 8:**
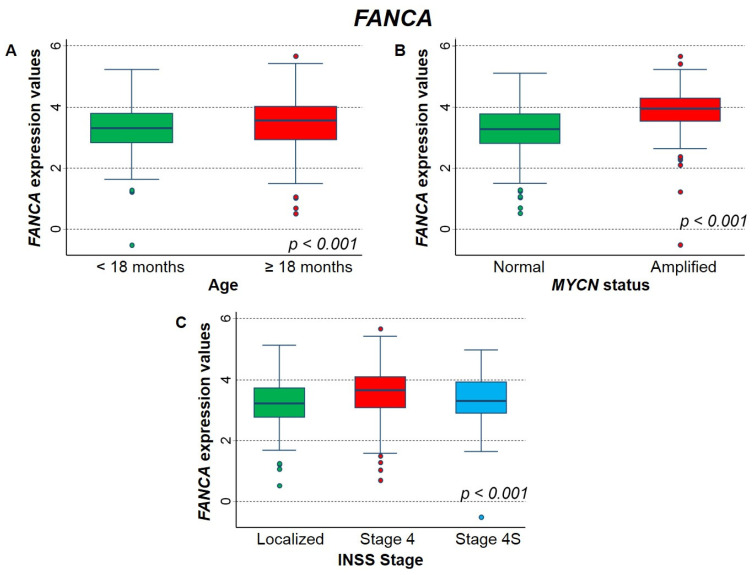
Association between *FANCA* gene expression and the main patient characteristics at diagnosis evaluated on the entire cohort. (**A**) Age; (**B**) *MYCN* status; (**C**) INSS stage.

Figure 8 shows the association between the expression of *FANCA* gene and the main patient characteristics at diagnosis. Higher values were significantly associated with higher age (Figure 8A), *MYCN* amplification (Figure 8B), and stage 4 disease (Figure 8C).

**Figure 9:**
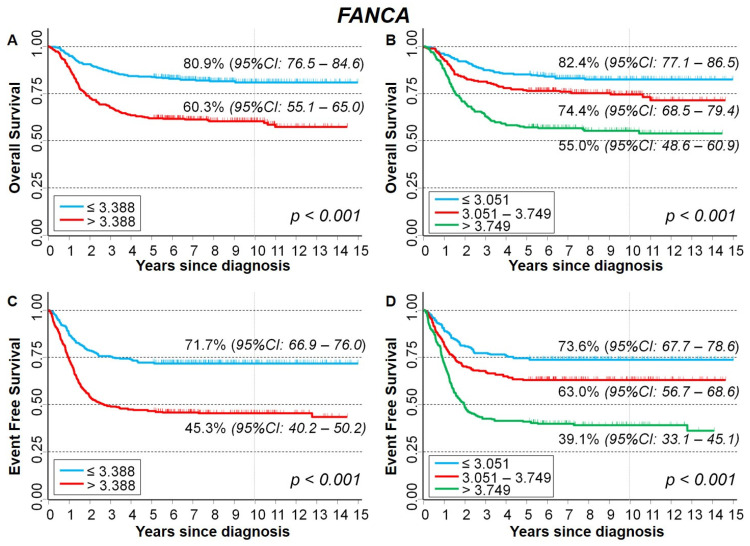
Kaplan–Meier survival curves of the whole cohort of 786 NB patients in relation to *FANCA* gene-expression values. (**A**) Overall survival, cut-off based on the median expression value; (**B**) Overall survival, cut-offs based on tertile expression values. (**C**) Event-free survival, cut-off based on the median expression value; (**D**) Event free survival, cut-offs based on tertile expression values. Ten-year survival estimates are displayed.

Figure 9 shows the Kaplan–Meier survival analysis in relation to *FANCA* expression levels. Higher values were significantly associated to a poorer survival for both OS and EFS. OS was 60.3% for values higher than the median expression level, and 80.9% under the median expression (Figure 9A). Stratification by tertile values highlighted a clear trend (OS = 55.0%, 74.4%, and 82.4%, respectively, Figure 9B). A similar pattern emerged for EFS (cut-off on median value: 45.3% vs. 71.7%, Figure 9C; cut-off on tertiles: 39.1%, 63.0%, and 73.6%, respectively, Figure 9D). The inverse association between *FANCA* expression levels and patient survival was clearly confirmed for EFS in multivariable Cox regression analysis, while for OS statistical significance was not reached (Table 4).

Stratification by *MYCN* status found a significant association between higher gene expression levels and poorer survival only in patients with normal *MYCN* status, both for OS and EFS (Supplementary Table S4). This association was also observed after stratification by stage at diagnosis (Supplementary Table S5) in patients with localized stage and in those with stage 4 disease, but not in the group with Stage 4S NB.

#### 2.3.3. Association between Patient Survival and Expression of MAPK3 Gene

**Figure 10:**
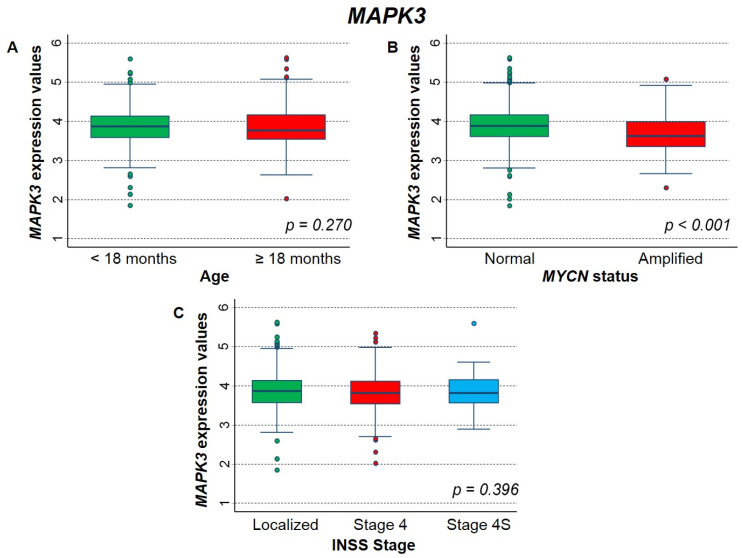
Association between *MAPK3* gene expression and the main patient characteristics at diagnosis evaluated on the entire cohort. (**A**) Age; (**B**) *MYCN* status; (**C**) INSS stage.

Figure 10 shows the association between *MAPK3* expression and the main patient characteristics at diagnosis. Higher values were significantly associated to normal *MYCN* status (Figure 10B), while no association emerged by age and stage at diagnosis (Figure 10A and Figure 10C, respectively).

**Figure 11:**
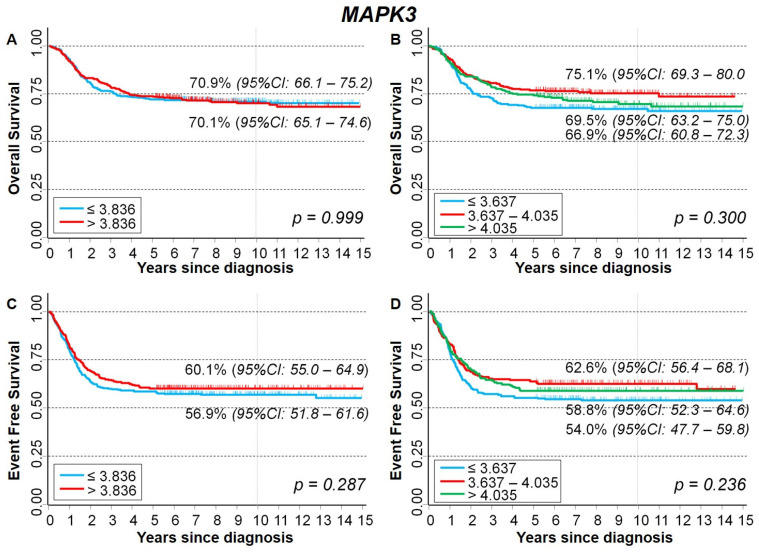
Kaplan–Meier survival curves of the whole cohort of 786 NB patients in relation to *MAPK3* gene-expression values. (**A**) Overall survival, cut-off based on the median expression value; (**B**) Overall survival, cut-offs based on tertile expression values. (**C**) Event-free survival, cut-off based on the median expression value; (**D**) Event-free survival, cut-offs based on tertile expression values. Ten-year survival estimates are displayed.

Kaplan–Meier survival analysis did not highlight any association between *MAPK3* gene expression and either OS (Figure 11A,B) or EFS (Figure 11C,D). In multivariable Cox regression analysis, values higher than the median were slightly associated with a poorer OS (HR = 1.4), but without any evidence of trend, while no association emerged for EFS (Table 5).

No association was observed between *MAPK3* expression levels and either OS or EFS after stratification by *MYCN* status (Supplementary Table S6). Stratification by stage (Supplementary Table S7) indicates a poorer OS for patients with localized disease and values below the first tertile. Conversely, in Stage 4S patients, a lower survival was associated to values above the median expression level for both OS and EFS, but without evidence of trend.

## 3. Discussion

The analysis of multiple samples from the same NB patient during illness allowed us to investigate genome changes during clinical progression from stage 4S to true stage 4 of this pediatric cancer. The examination of the a-CGH data in combination with WES results allowed us to recognize some chromosomal and gene-driver alterations that occurred during the time elapsed between diagnosis and disease recurrence. Studying chromosomal imbalances in the number of copies in the NB material at onset (stage 4S) and after progression to true stage 4, and correlating them with the somatic variants, we gained new insights into the molecular mechanisms underlying the tumor progression. Some chromosomal alterations analyzed at the disease progression were atypical (loss 12p13.31, loss 16p11.2, and loss 16q11.2–q24.3), while the loss 4p16.3–p12 was already known for its nasty potential [36]. The loss 12p13.31 contained the *KLRB1* gene encoding the CD161 receptor of NK cells, and it is currently thought that this gene affects tumorigenesis by regulating the cytotoxicity of NK cells in cancer [27]. Interestingly, NB tumors displaying a low NK-cell density are devoid of DC cells and strongly associated with poor prognosis [9]. Notably, the loss 16p11.2 is significantly enriched in NB cases and the disruption of this region disregulates neurodevelopmental pathways that influence NB [28]. The effects of this deletion may be due to deregulation of the MAPK/ERK pathway caused by the loss of the *MAPK3* gene [28]. The loss 16q was particularly remarkable since it contained *FANCA*, a gene involved in DNA repair [29], and *CHD9*, a gene involved in chromatin regulation in NB [30]. Moreover, it has been demonstrated that the downregulation of *CHD9* is correlated with metastatic spread to the bone and low survival rate of NB patients [30]. To understand if the loss of *KLRB1, FANCA*, and *MAPK3* genes were relevant for NB progression, we analyzed in silico their expression and outcome in 786 NB patients, using the dataset summarized by Cangelosi et al. [37]. We chose these genes among others because *KLRB1*, *MAPK3*, and *FANCA* genes showed some importance in the development of different tumors [27,28,29]. In particular, loss of the *FANCA* gene leads to chromosomal instability promoting neoplastic transformation [38]. These analyses revealed that *KLRB1* gene expression was associated with both OS and EFS, as higher levels of expression corresponded to better survival. However, the effect diminished and was no longer significant when correcting for *MYCN* amplification (MNA), age at diagnosis, and stage. In the stratified analysis, the association was present only in patients with normal *MYCN* status, as is the case of our patient. In both localized and stage 4, the association disappeared correcting for MNA and age at diagnosis. In stage 4S, no association was observed, but the sample size was very low. As for the *FANCA* gene, an inverse association was observed with both OS and EFS, as high levels of expression corresponded to low survival. Stratifying by MNA, the association was still evident only in NB patients with normal *MYCN* status. Contrary to what we observed by analyzing the expression of *KLRB1* and *FANCA* genes, the expression of the *MAPK3* gene did not display evident associations with survival of NB patients. The data are quite indicative of the potential role of the *KLRB1* and *FANCA* genes’ expression in the prognosis of NB. Since high levels of expression of the *KLRB1* gene seem to correspond to better survival of NB patients with normal *MYCN* status, the loss of *KLRB1*—and therefore, the decrease/lack of the specific receptor—probably leads to a failure of NK cells to recognize NB cells, thus promoting tumor growth and progression in the absence of MNA, as in the present case. A novel role of the *FANCA* gene other than in the DNA repair pathway has recently been described [33]. A high expression of the *FANCA* gene determines a worse prognosis in the chronic lymphocytic leukemia as it impairs p53 function [39]. Our results suggest that this probably also applies to NB, even if it was not the case of our patient, who did not benefit from the tumor loss of *FANCA* gene, possibly because his clinical picture was too compromised.

Analyzing the WES data, we noticed that most of the clones arisen during the progression of the disease were enriched with new somatic variants. The participation of the chromosomal copy number aberrations (CNAs) suggests an early generation of clonal diversity that was primarily driven from imbalances affecting the disadvantageous alleles. Additional newly emerging somatic SNVs could have helped determine the progression. Among the gene mutations analyzed at the time of diagnosis, we found a pathogenic variant of *FGFR1* gene, whose mutations were described to be associated with NB aggressiveness [4,6,40]. At the time of progression only, we identified the frameshift mutation of *CASZ1* gene that functions as a tumor suppressor in NB contributing to cell-cycle deregulation [41]. The missense mutation of the *PBX2* gene could contribute to tumor progression by controlling the gene regulatory networks involved in cell proliferation, apoptosis, cell cycle, epithelial-mesenchymal transition (EMT), invasion, and metastasis, as well as the stemness of cancer cells [42]. The frameshift mutation of *DMBT1* gene at progression could be important, as it has been shown to contribute to the development of a small fraction of NB [43]. Finally, we found in all samples analyzed at onset of disease and at time of progression the same missense variant in the *PTPN11* gene. The *PTPN11* gene encodes for the tyrosine phosphatase SHP2, and it is considered a driver gene for NB [4,6]. Furthermore, evidence in zebrafish demonstrated that *PTPN11* promoted NB tumorigenesis activating the RAS/MAPK pathway [44]. Therefore, the presence of the *PTPN11* gene mutation at onset, maybe together with the other important mutation on the *FGFR1* gene, could have been decisive to cause progression and relapse. We also identified SNVs in genes that are infrequently mutated and not characteristic of NB, such as the mutation of the *TYW1* gene that encodes for an iron-sulfur protein involved in neuronal function [45]. Although these SNVs are not currently associated with NB, they could be revealed as determinant in the future. This is in accordance with the literature, which suggests that tumor genomes are characterized by a few genes frequently mutated among patients, and by a much larger number of more rarely mutated genes contributing to the onset and progression of the disease [46]. Moreover, at the time of progression, we identified CNVs within known driver genes of NB, such as *BRAF* and *NF1*, involved in the RAS/MAPK pathway, and a small deletion within the *ATRX* gene. Therefore, we have identified mutations responsible for acquired resistance, and in genes involved in RAS/MAPK pathway, typical of relapsed NB [19]. It has recently shown that 68% of *ATRX* mutations are multi-exon deletions, and that they are associated with poor prognosis of NB patients [47]. In conclusion, we disclosed during the progression to true stage 4 mutations of some genes that identify an aggressive NB. Currently, there is a significant interest in identifying aberrant signaling pathways and RAS/MAPK activating mutations, like those of *PTPN11* and *FGFR1* genes detected in the present case, that could be therapeutically targeted for relapsing NB. Preclinical NB studies have demonstrated that pharmacological inhibition of SHP2 with SHP099, RMC-4550, and II-B08 in combination with trametinib, vemurafenib, and ulixertinib inhibitors suppresses the growth and the downstream RAS/MAPK signaling in NB cells in vitro and in vivo [48]. FGFR1 is a tyrosine kinase receptor whose inhibitors have been used extensively in Phase I/II clinical trials for treatment in cancers presenting *FGFR1* mutation [49]. It was noted that the combination of AZD4547 and GDC0941 inhibits the activating effects of the *FGFR1* mutation in NB cells in vitro [50].

It is remarkable that the patient disclosed a NCA profile in primary tumor, while at progression he displayed a SCA profile with 4 chromosome partial losses and 12 numerical whole-chromosome abnormalities. The high rate of chromosomal instability and the numerous acquired mutations found in the tumor that progressed to true stage 4 correlated with the unfavorable clinical picture, with the rapid observed tumor progression, and with the dramatic decline of patient’s conditions, that led him rapidly to death. The results of this study support the possibility of obtaining a better prognosis for patients with NB by systematically carrying out an accurate genome analysis at onset. We also confirm our previous observations about genetic analysis carried on DNA obtained from plasma NB-derived exosomes [26] that are fully informative, as well as on DNA extracted from a tumor biopsy, but with a less invasive way of extraction for the patient.

## 4. Materials and Methods

### 4.1. Patient Information and Sample Collection

A 10-month-old boy was referred to our examination for general asthenia. Ultrasonography showed an abdominal solid mass confirmed by a CT scan that identified a solid mass in the left adrenal gland (9 × 9 × 8 cm), with bone marrow metastases < 10%. The pathological diagnosis was a stroma-poor, poorly differentiated NB without *MYCN* amplification and carrying numerical whole-chromosome alterations. The patient was therefore considered as stage 4S NB [20,21,22,23,24,25] with low risk [7], and after informed consent, enrolled in the LINES (group four) European SIOPEN protocol with no treatment expected. In the following twelve months, a spontaneous gradual reduction in the mass volume and the normalization of the bone marrow were observed. One year after the diagnosis of stage 4S NB, the patient showed a marked lack of appetite, irritability, and a right latero-cervical lymph-adenomegaly. Neck and abdomen ultrasound was performed, showing an increase in the size of the adrenal mass from 5 to 7 cm. Bone marrow aspirate and CT of neck, thorax, and abdomen showed massive bone marrow infiltration, expansion of the left adrenal lodge (4.5 × 9.7 × 7.5 cm) with consequent downwards dislocation of the left kidney, and right supraclavicular lymph node metastasis. The surgical biopsy of the right supraclavicular neoformation was diagnosed as stroma-poor, poorly differentiated progression to stage 4 NB. The patient was therefore considered as high-risk NB [7] and treated—after a new informed consent—according to the European HR-NBL1/SIOPEN protocol [51]. After eleven months, the patient relapsed to skull, bone marrow, and liver, with further progression in the known sites, and he rapidly became worse during the next four months, until death.

Primary NB tissues at the time of diagnosis and at the time of disease progression were obtained before treatment. Tumor samples were stored in the BIT-NB (Biobank Integrated Tumor-Neuroblastoma) Tissue Section of IRCCS G. Gaslini, Genova, Italy. NB samples content was confirmed by review of hematoxylin and eosin-stained tumor sections by the local pathologists.

Genomic DNA (gDNA) from peripheral blood lymphocytes and tumor DNAs were extracted using QIAamp DNA Extraction Kit (Qiagen, Hilden, Germany), according to the manufacturer’s instructions.

### 4.2. Genomic Profile Analysis

DNAs from the primary tumor at onset and from the tumor at disease progression were tested by high-resolution oligonucleotide a-CGH using the 4x180 K Kit (Agilent Technologies, Santa Clara, CA, USA) with a mean resolution of approximately 25 kb [52]. Each hybridization produced a pair of 16-bit images, which were processed using the Agilent Feature Extraction 10.5 Software. The data were analyzed using the Genomic Workbench 7.0.40 software (Agilent) and the altered chromosomal regions and breakpoints events were detected using ADM-1 (threshold 10) with 0.5 Mb window size to reduce false positives [53]. Amplifications were defined at loci with log2 ratio ≥ 2, and loci with log2 ratio ≥ 3.5 were considered as high level of amplifications. Chromosome positions were determined using GRCh38/hg19 (UCSC Human Genome Browser, http://genome.ucsc.edu, February 2009, NCBI Build 37.1, accessed on 20 January 2022). The chromosomal copy number variations present in the Database of Genomic Variants (DGV: http://projects.tcag.ca/variation/, accessed on 14 February 2022) were taken into consideration only with a frequency < 5%. The raw data are stored in the BIT-NB (Biobank Integrated Tumor-Neuroblastoma) Genomic Section of IRCCS G. Gaslini.

Written informed consent was obtained from the parents in accordance with the Declaration of Helsinki to report the case of their child. This study was approved by the Italian Institutional Ethics Committee (Measure num. 270/17 related to the clinical study protocol IGG-NCA-AP-2016).

### 4.3. Exo-DNA Purification and Quantification

Blood samples were collected in EDTA tubes and plasma was separated from blood by centrifugation at 1600× *g* for 10 min at 4 °C twice, followed by aliquoting and freezing at −80 °C [54]. We also isolated and lysed exosomes from the plasma NB samples collected at onset of disease and at the time of progression, then we extracted exo-DNA and evaluated its quality and quantity as previously described [26]. Exosomes for immunofluorescence analysis were resuspended in 100 µL 1× PBS for Cytospin cell preparation and incubated with anti-GD2 mAb (1:500; secreted from hybridoma cell line 14.G2, a generous gift by R.A. Reisfeld, The Scripps Research Institute, La Jolla, CA, USA), followed by a specific secondary antibody conjugated with Alexa 488 (green). Immunofluorescence analysis was performed as previously described [55]. Results were photographically documented using fluorescence microscope Axio Imager M2 (Carl Zeiss, Oberkoche, Germany).

### 4.4. Library Construction and Whole Exome Sequencing

Quality control of gDNA was performed using the DNA Broad Range Qubit Assay (ThermoFisher, Waltham, MA, USA) and the 4150 TapeStation System (Agilent Technologies). Samples were sheared to 300 bp using the Covaris S220 instrument (Covaris, Woburn, MA, USA) according to the manufacturer’s protocol. Library was constructed using the KAPA Hyper Prep (Roche, Basel, Switzerland). Exo-DNA at tumor onset and at progression were adapter ligated with xGen Dual Index UMI Adapters (IDT, Coralville, IA, USA). Library quality control was carried out using the DNA Broad Range Qubit Assay (ThermoFisher) and the 4150 TapeStation System (Agilent Technologies). Exomes were captured by the Twist Human Core Exome + RefSeq (Twist Bioscience, San Francisco, CA, USA) following the manufacture’s indications. Enriched libraries were quantified by real-time PCR using the KAPA Library Quantification Kit for Illumina platforms on the QuantStudio3 Real-Time PCR Systems (ThermoFisher) and polled at equimolar concentrations. Sequencing was performed on the Novaseq 6000 platform (Illumina, San Diego, CA, USA) using the 150 bp paired-end mode.

CNVs in exo-DNA and in tumor samples were identified using the tool EXCAVATOR2 with default setting, pairing each tumor DNA and exo-DNA with the corresponding gDNA sample, as previously described [26].

### 4.5. Bioinformatics Pipeline

Reads were aligned to the reference human genome sequence version GRCh38/hg38 using BWA-MEM v0.7.15 (https://arxiv.org/abs/1303.3997, accessed on 10 March 2022). For the exosome and tumor samples processed with UMI, fgbio v1.1.0 was used to first integrate the UMI information into the BAM files and to group the reads by UMI using the “Annotate Bam with Umis” and “Group Reads by Umi” functions, respectively. Consensus reads based on UMI were then generated through the “Call Molecular Consensus Reads” function (using parameters: --error-rate-post-umi = 30 and –mim-reads = 1). The consensus reads were then re-mapped to the human reference’s genome. All generated BAM files were cleaned by local realignment around insertion–deletion sites, duplicate marking (only for the samples processed without UMI), and recalibration using Genome Analysis Toolkit v3.8.1.6. Overlapping regions of the BAM file were clipped using BamUtil v1.4.14 to avoid counting multiple reads representing the same fragment. CallableLoci in GATK v3.8 was used to identify callable regions of the target (genotypability), with minimum read depths of 3 and 10. CollectHsMetrics by Picard v2.17.10 was used to calculate fold enrichment and FOLD 80 penalty values to determine enrichment quality. Somatic variant calling was then performed with Mutect2 (GATK v4.1.9.0) in the “tumor with matched normal” mode, considering as “tumor” the exo-DNA and tumor samples, and as “normal” the germline genomic DNA (gDNA). As a preliminary step, a “panel of normal” VCF file was created using all the germinal gDNA samples to further refine the somatic analysis. Variants were then filtered by quality (filter PASS) and target design region. Annotation and prioritization of variants was carried out using VarSeq software (GoldenHelix). The annotation process included databases of somatic mutations (COSMIC v90, TCGA Variants 25 March 2019, GHI, ICGC Simple Somatic Mutations 28, GHI, CIViC—Variant Clinical Evidence Summaries 1 June 2020, WUSTL, CIViC—Genes 1 June 2020, WUSTL), the RefSeq Genes 109 Interim v2.1 NCBI and the dbSNP 153 NCBI databases.

### 4.6. Data Source

Information about the expression of *KLRB1*, *FANCA*, and *MAPK3* genes was obtained from the large dataset summarized by Cangelosi et al. [37], which collects 13,698 gene-expression profiles from 786 NB primary tumors. Data were obtained by integrating gene-expression values from three different platforms, and included clinical characteristics of patients at diagnosis (age, stage, amplification of the proto-oncogene *MYCN*), survival time, and related events (disease recurrence or death for any cause).

Supplementary Table S8 resumes the main characteristics of the considered patients: 337 (43%) were ≥18 months of age, 153 (20%) had *MYCN* amplification and 373 (48%) were diagnosed in localized stages. These latter included 18% stage 1, 16% stage 2, and 13% stage 3, while 320 patients had stage 4 disease (41%) and 92 (12%) had a stage 4S NB. The number of observed events was 320 and the observed deaths were 229. Seventeen patients had no information about the disease recurrence. More details are available on the related publication [37].

### 4.7. Statistical Analysis

Association between patient survival and the expression of the three considered genes was assessed by the Kaplan–Meier method. The patient cohort was split into both two and three groups of approximately equal sample size based on median and tertiles values. Overall survival (OS) was calculated from the date of diagnosis to the date of death or to the last contact for censored data. Event-free Survival (EFS) was calculated from the date of diagnosis to the first occurrence between death and disease recurrence. The related 95% confidence intervals (95% CI) of survival estimates were obtained by the Kalbfleisch and Prentice method [56]. No information was available about the occurrence of secondary tumors. The log-rank test and the log-rank test for trend, when appropriated, were applied to compare Kaplan–Meier curves, while the Cox regression model was fitted to adjust for the confounding effect of the potential prognostic factors at diagnosis, available in the dataset, i.e., age (<18 months vs. >18 months), *MYCN* status, and INSS stage [37]. Subgroup analyses included stratification by *MYCN* status and stage at diagnosis.

All tests were two-sided, and a *p*-value < 0.05 was considered as statistically significant. All analyses were performed by STATA for Windows statistical package (release 13.1, Stata Corporation, College Station, TX, USA).

## 5. Conclusions

We confirmed our previous observations on DNA extracted from plasma NB-derived exosomes that can give a complete genetic information as a normal tumor biopsy, but with higher advantage for the patient, because this sampling can be repeated over time. WES analysis of exo-DNA at onset can be predictive of possible tumor progression and relapse. Integrated study of the mutational profile by WES and of chromosomal aberrations by a-CGH at diagnosis can provide information on future clonal dynamics that allow better monitoring the course of the disease. Furthermore, we identified a possible oncosuppressive and oncogenic role, respectively, for *KLRB1* and *FANCA* genes in NB progression.

## Figures and Tables

**Table 1:** Patient’s characteristics and tumor DNA genomic profile analysis.

	INRG Stage	Age (Months)	*MYCN* Status	Genomic Profile by Array-CGH	Chromosome Alterations
Tumor onset	MS	10	Single copy	Numerical	+4, +6, +7, −9, −10, −11, +13, −14, +15, +17, −X, +Y
Tumor progression	M	23	Single copy	Segmental	4p−, +6, +7, −9, −10, −11, −12, 12p−, +13, −14, +15, 16p−, 16q−, +17, −X, +Y

**Table 2:** Genes displaying copy number variations (CNVs).

Sample	Chr	Gene	Start	End	CNV Length (bp)	Copy Number *
Tumor onset	None					
Exo-Onset	None					
Exo-progression	Chr 7	*BRAF*	127,186,913	143,594,351	16,407,439	3
	Chr 17	*NF1*	26,944,359	36,166,563	9,222,205	3
	Chr X	*ATRX*	62,470,621	155,705,476	93,234,856	1

* The standard copy number is 2.

**Table 3:** Survival of the whole cohort of patients with NB in relation to *KLRB1* gene-expression values, evaluated by the multivariable Cox regression model.

	Overall Survival			Event Free Survival		
Gene Expression	HR	95% CI	*p*	HR	95% CI	*p*
Median			0.053			0.069
≤1.377 (reference)	1	-		1	-	
>1.377	0.74	0.55–1.0		0.80	0.62–1.0	
Tertiles			0.046 *			0.052 *
≤0.832 (reference)	1	-		1	-	
0.832–1.807	0.99	0.72–1.35		0.93	0.71–1.2	
>1.807	0.65	0.45–0.96		0.74	0.54–1.0	

HR: Hazard ratio. Multivariable analysis: HRs are adjusted by *MYCN* status, age, and stage at diagnosis. * Test for trend.

**Table 4:** Survival of the whole cohort of patients with NB in relation to *FANCA* gene-expression values, evaluated by the multivariable Cox regression model.

	Overall Survival			Event Free Survival		
Gene Expression	HR	95% CI	*p*	HR	95% CI	*p*
Median			0.070			<0.001
≤3.388 (reference)	1	-		1	-	
>3.388	1.3	0.98–1.8		1.6	1.3–2.1	
Tertiles			0.054 *			<0.001 *
≤3.051 (reference)	1	-		1	-	
3.051–3.749	1.3	0.88–1.9		1.4	1.1–2.0	
>3.749	1.4	1.0–2.1		1.9	1.4–2.6	

HR: Hazard ratio. Multivariable analysis: HRs are adjusted by *MYCN* status, age, and stage at diagnosis. * Test for trend.

**Table 5:** Survival of the whole cohort of patients with NB in relation to *MAPK3* gene-expression values, evaluated by the multivariable Cox regression model.

	Overall Survival			Event Free Survival		
Gene Expression	HR	95% CI	*p*	HR	95% CI	*p*
Median			0.021			0.704
≤3.836 (reference)	1	-		1	-	
>3.836	1.4	1.0–1.8		1.0	0.83–1.3	
Tertiles			0.094 *			0.728 *
≤3.637 (reference)	1	-		1	-	
3.637–4.035	1.0	0.74–1.4		0.97	0.74–1.3	
>4.035	1.3	0.96–1.8		1.1	0.80–1.4	

HR: Hazard ratio. Multivariable analysis: HRs are adjusted by *MYCN* status, age, and stage at diagnosis. * Test for trend.

## Data Availability

Data are contained within the article or Supplementary Material.

## Supplementary Materials

The following supporting information can be downloaded at: www.mdpi.com/article/10.3390/ijms23126513/s1.

## Abbreviations

NB	Neuroblastoma
INRG	International Neuroblastoma Risk Group
INSS	International Neuroblastoma Staging System
LINES	European Low and Intermediate Risk Neuroblastoma Study
SIOPEN	International Society of Pediatric Oncology European Neuroblastoma
*MYCN*	v-myc myelocytomatosis viral related oncogene, neuroblastoma derived
MNA	*MYCN* amplification
TME	tumor microenvironment
DCs	dendritic cells
NK	natural killer cells
a-CGH	array-Comparative Genome Hybridization
SCA	segmental chromosomal alteration
NCA	numerical chromosomal alteration
Chr	Chromosome
gDNA	genomic DNA
exo-DNA	exosomal DNA
EMT	Epithelial-Mesenchymal Transition
WES	Whole Exome Sequencing
SNV	Single-Nucleotide Variant
CNV	Copy Number Variation
CNAs	Copy Number Aberrations
VAF	Variant Allele Frequency
VCF	Variant Call Format
COSMIC	Catalogue of Somatic Mutations in Cancer
