# Supplementary material for: Genomic Analysis Made It Possible to Identify Gene-Driver Alterations Covering the Time Window between Diagnosis of Neuroblastoma 4S and the Progression to Stage 4
Source: Int J Mol Sci. 2022 Jun 10;23(12):6513. doi: 10.3390/ijms23126513 (PMC9224358; doi:10.3390/ijms23126513)
Supplement: Supplementary file 1 [file ijms-23-06513-s001.zip › ijms-1748841-supplementary.pdf]

**Supplementary Material, Ognibene M. et al.**

**Supplementary Table S1.** Pathogenic or likely pathogenic SNVs *

| **NB driver genes** | | **Variant type** |
|---|---|---|
| *CASZ1* c.4966 del G | | frameshift |
| *PBX2* c.41 G>A | | missense |
| *FGFR1* c.1625 del A | | frameshift |
| *DMBT1* c.7217 del C | | frameshift |
| *PTPN11* c.1508 G>T | | missense |
| **Other mutated genes** | | |
| *HTR1D* c.680 G>A | | missense |
| *TYW1* c.1273 C>T | | missense |
| *LRP1B* c.13686 T>G | | stop gained |
| *MAST2* c.1301 C>A | | missense |
| *GNG12* c.202 del A | | frameshift |
| *BARHL2* c.865 C>T | | missense |
| *KCNA10* c.352 de lA | | frameshift |
| *KCNA10* c.352 A>G | | missense |
| *INKA2* c.559 del G | | frameshift |
| *GON4L* c.5490 delA | | frameshift |
| *FCRL6* c.1302 C>A | | stop gained |
| *ADCY10* c.4518 C>A | | splice region |
| *TDRD5* c.1907 del T | | frameshift |
| *CEP350* c.4361 A>G | | missense |
| *RBBP5* c.997 G>A | | missense |
| *PKDCC* c.254G>T | | missense |
| *ATP6V1B1* c.33 del G | | frameshift |
| *DOK1* c.245 del C | | frameshift |
| *AFF3* c.1696 del G | | frameshift |
| *EN1* c.820 G>A | | missense |
| *MYO7B* c.6313 C>T | | stop gained |
| *SAP130* c.1787 T>A | | missense |
| *PTPN18* c.826 C>T | | stop gained |
| *MCM6* c.1825 G>T | | missense |
| *VIL1* c.2035 C>T | | stop gained |
| *TUBA4A* c.355 C>T | | splice region |
| *TMEM198* c.653 G>T | | missense |
| *MTMR14* c.1490 del C | | frameshift |
| *ZBTB47* c.1634 A>G | | missense |
| *LAMB2* c.2882 C>T | | missense |
| *IFRD2* c.955_956 AA>CC | | missense |
| *PBRM1* c.3520 T>C | | missense |
| *SI* c.1882 C>A | | missense |
| *EVC2* c.24 del G | | frameshift |
| *CRMP1* c.381+4323 A>G | | initiator codon |
| *SRD5A3* c.312 del T | | frameshift |
| *PARM1* c.784 A>C | | missense |
| *PRDM8* c.822 C>T | | splice region |
| *ANK2* c.10367 dup G | | frameshift |
| *CCNA2* c.293 C>T | | missense |
| *PCDH10* c.49 T>C | | missense |

| | | |
|---|---|---|
| *AGXT2* c.1421 G>A | | missense |
| *NUP155* c.166 A>C | | missense |
| *ITGA1* c.87 C>A | | missense |
| *IK* c.814 C>G | | missense |
| *PDE6A* c.2381 A>G | | missense |
| *TCOF1* c.2873 del C | | frameshift |
| *NPM1* c.527 A>G | | missense |
| *NSD1* c.2903 del A | | frameshift |
| *SLC22A23* c.1864 A>T | | frameshift |
| *SLC22A23* c.932 del C | | missense |
| *ARFGEF3* c.2096 C>A | | missense |
| *ARFGEF3* c.4207 del G | | frameshift |
| *ARFGEF3* c.4207 G>T | | missense |
| *SASH1* c.1756 del A | | frameshift |
| *SYNE1* c.11394 del A | | frameshift |
| *ANLN* c.523 C>T | | missense |
| *SEPTIN14* c.775 del A | | frameshift |
| *SAMD9* c.3559 del A | | frameshift |
| *KLHDC10* c.604 A>G | | missense |
| *PLXNA4* c.5573 G>A | | missense |
| *ADAM9* c.883 C>T | | missense |
| *PARP10* c.1829 A>G | | missense |
| *FAM189A2* c.1157 C>T | | missense |
| *C5* c.3479 C>T | | missense |
| *HMCN2* c.8956 G>A | | missense |
| *FUBP3* c.566 A>G | | missense |
| *CEL* c.2171 del C | | frameshift |
| *TRAF2* c.26 del C | | frameshift |
| *ITGA8* c.1344 C>T | | splice region |
| *ADRA2A* c.1019 A>T | | missense |
| *TRPM5* c.2906 T>G | | missense |
| *CARS1* c.1265 del A | | frameshift |
| *EIF3F* c.1034 A>G | | missense |
| *MRPL48* c.222 del A | | frameshift |
| *EED* c.770 A>T | | missense |
| *JHY* c.2216 del A | | frameshift |
| *CACNA1C* c.2336 C>T | | missense |
| *STK38L* c.710 G>T | | missense |
| *CAND1* c.674 del A | | frameshift |
| *BTBD11* c.2570 A>G | | missense |
| *HNF1A* c.1463 C>A | | missense |
| *MYCBP2* c.1173 C>T | | splice region |
| *CCDC168* c.17048 del A | | frameshift |
| *CCDC168* c.12092 del A | | frameshift |
| *SOX1* c.342 G>A | | splice region |
| *CUL4A* c.1952 G>T | | missense |
| *ABCD4* c.868 G>A | | missense |
| *RTF1* c.1098 G>A | | splice region |
| *TBC1D24* c.1531 G>A | | missense |
| *SRL* c.734 G>A | | missense |
| *GLYR1* c.874 A>G | | missense |
| *ITGAM* c.856 G>T | | missense |
| *ZNRF1* c.199 del G | | frameshift |
| *KIAA0513* c.73 G>T | | stop gained |

| | | |
|---|---|---|
| *TRPV3* c.353 G>A | | missense |
| *ENO3* c.313 A>C | | missense |
| *DHX33* c.1393 C>G | | missense |
| *NLGN2* c.1967 A>G | | missense |
| *SLC35G6* c.997_998 GA>AC | | missense |
| *DHRS7C* c.141 A>G | | splice region |
| *KSR1* c.146 A>G | | missense |
| *MED1* c.1172 C>T | | missense |
| *NR1D1* c.610 C>T | | missense |
| *OTOP2* c.1456 del A | | frameshift |
| *CCDC137* c.799 C>T | | stop gained |
| *ZBTB7C* c.1435 C>G | | missense |
| *ATP8B1* c.2441 A>G | | missense |
| *RTTN* c.2303 dup A | | frameshift |
| *SF3A2* c.337 G>A | | missense |
| *ARHGEF18* c.434 A>T | | missense |
| *AP1M1* c.546+1201 G>T | | stop gained |
| *MYO9B* c.4154 del A | | frameshift |
| *ZNF682* c.146 delA | | frameshift |
| *TDRD12* c.3378 dup G | | frameshift |
| *KIRREL2* c.606 del T | | frameshift |
| *LTBP4* c.3958 G>T | | missense |
| *CTU1* c.166 G>A | | missense |
| *ZSCAN18* c.1418 dup G | | frameshift |
| *RTF2* c.340 C>A | | missense |
| *TIAM1* c.3284 A>G | | missense |
| *RIPK4* c.27 G>T | | missense |
| *TRPM2* c.1273 G>A | | missense |
| *FOXRED2* c.1663 C>T | | missense |
| *BAIAP2L2* c.1392 del C | | frameshift |
| *NPTXR* c.1 C>A | | initiator codon |
| *GTPBP6* c.170 T>A | | missense |
| *TAB3* c.1772 C>T | | missense |
| *PPP1R2C* c.202 G>A | | missense |
| *SASH3* c.14 A>C | | missense |

Legend: * Pathogenicity of SNVs was checked using multiple web-based applications evaluating DNA sequence variants for their disease-causing potential

Mutation detected only in tumor onset DNA

Mutation detected only in exo-DNA at onset

Mutation detected only in exo-DNA at progression

Concordant mutation in tumor DNA and in exo-DNA at progression

Concordant mutation in tumor DNA, in exo-DNA at onset and in exo-DNA at progression

**Supplementary Table S2.** Survival of 786 NB patients in relation to *KLRB1* expression levels by *MYCN* status.

| Overall Survival | **N/D** | **OS** | **95%CI** | ***p*** |
|---|---|---|---|---|
| *MYCN normal* | | | | |
| Cut-off based on the median value | | | | *0.002* |
| ≤ 1.559 | 314/74 | 77.1 | 72.0 – 81.4 | |
| > 1.559 | 315/45 | 84.6 | 79.7 – 88.4 | |
| Cut-offs based on tertile values | | | | *< 0.001* |
| ≤ 1.174 | 209/58 | 72.2 | 65.5 – 77.9 | |
| 1.174 – 1.945 | 210/32 | 85.1 | 79.3 – 89.5 | |
| > 1.945 | 210/29 | 85.2 | 79.2 – 89.6 | |
| *MYCN amplified* | | | | |
| Cut-off based on the median value | | | | *0.537* |
| ≤ 0.391 | 76/54 | 28.5 | 18.7 – 39.0 | |
| > 0.391 | 77/54 | 29.9 | 20.1 – 40.2 | |
| Cut-offs based on tertile values | | | | *0.803* |
| ≤ 0.152 | 51/36 | 29.0 | 17.2 – 41.8 | |
| 0.152 – 0.825 | 51/31 | 39.2 | 26.0 – 52.2 | |
| > 0.825 | 51/41 | 19.6 | 10.1 – 31.4 | |

| Event Free Survival | **N/E** | **EFS** | **95%CI** | ***p*** |
|---|---|---|---|---|
| *MYCN normal* | | | | |
| Cut-off based on the median value | | | | *0.027* |
| ≤ 1.559 | 307/116 | 62.5 | 56.8 – 67.7 | |
| > 1.559 | 307/91 | 70.3 | 64.9 – 75.1 | |
| Cut-offs based on tertile values | | | | *0.001* |
| ≤ 1.174 | 203/87 | 57.6 | 50.5 – 64.1 | |
| 1.174 – 1.945 | 206/62 | 69.9 | 63.1 – 75.7 | |
| > 1.945 | 205/58 | 71.7 | 65.0 – 77.3 | |
| *MYCN amplified* | | | | |
| Cut-off based on the median value | | | | *0.390* |
| ≤ 0.391 | 75/56 | 24.2 | 14.9 – 34.9 | |
| > 0.391 | 76/55 | 27.6 | 18.2 – 37.9 | |
| Cut-offs based on tertile values | | | | *0.837* |
| ≤ 0.152 | 51/39 | 21.9 | 11.1 – 34.9 | |
| 0.152 – 0.825 | 50/31 | 38.0 | 24.8 – 51.1 | |
| > 0.825 | 50/41 | 18.0 | 8.9 – 29.7 | |

N/D = Number of patients/ Deaths. OS = Ten-year Overall Survival, N/E Number of patients/ Events. EFS = Ten-year Event Free Survival, 95%CI: 95% confidence interval.

**Supplementary Table S3.** Survival of 786 NB patients in relation to *KLRB1* expression levels by INSS stage.

| Overall Survival | N/D | OS | 95%CI | *p* |
|---|---|---|---|---|
| *Localized stage* | | | | |
| Cut-off based on the median value | | | | *0.007* |
| ≤ 1.556 | 186/25 | 86.5 | 80.7 – 90.7 | |
| > 1.556 | 187/10 | 94.6 | 90.2 – 97.1 | |
| Cut-offs based on tertile values | | | | *0.002* |
| ≤ 1.196 | 124/21 | 83.1 | 75.2 – 88.6 | |
| 1.196 – 1.952 | 124/7 | 94.2 | 88.3 – 97.2 | |
| > 1.952 | 125/7 | 94.4 | 88.6 – 97.3 | |
| *Stage 4* | | | | |
| Cut-off based on the median value | | | | *< 0.001* |
| ≤ 1.120 | 160/107 | 33.6 | 26.4 – 41.0 | |
| > 1.120 | 160/76 | 51.3 | 42.7 – 59.2 | |
| Cut-offs based on tertile values | | | | *< 0.001* |
| ≤ 0.468 | 106/73 | 31.9 | 23.3 – 40.9 | |
| 0.468 – 1.582 | 107/61 | 44.4 | 34.7 – 53.6 | |
| > 1.582 | 107/49 | 50.7 | 39.8 – 60.6 | |
| *Stage 4S* | | | | |
| Cut-off based on the median value | | | | *0.113* |
| ≤ 1.474 | 46/8 | 81.4 | 65.8 – 90.4 | |
| > 1.474 | 46/3 | 93.5 | 81.1 – 97.9 | |
| Cut-offs based on tertile values | | | | *n.e.* |
| ≤ 0.882 | 30/5 | n.e. | n.e. | |
| 0.882 – 1.800 | 31/5 | n.e. | n.e. | |
| > 1.800 | 31/1 | n.e. | n.e. | |

| Event Free Survival | N/E | EFS | 95%CI | *p* |
|---|---|---|---|---|
| *Localized stage* | | | | |
| Cut-off based on the median value | | | | *0.011* |
| ≤ 1.556 | 184/54 | 70.7 | 63.5 – 76.7 | |
| > 1.556 | 180/32 | 82.2 | 75.8 – 87.1 | |
| Cut-offs based on tertile values | | | | *0.004* |
| ≤ 1.196 | 122/41 | 66.4 | 57.3 – 74.0 | |
| 1.196 – 1.952 | 122/24 | 80.3 | 72.1 – 86.4 | |
| > 1.952 | 120/21 | 82.5 | 74.4 – 88.2 | |
| *Stage 4* | | | | |
| Cut-off based on the median value | | | | *0.002* |
| ≤ 1.120 | 157/112 | 29.1 | 22.2 – 36.4 | |
| > 1.120 | 157/94 | 40.1 | 32.4 – 47.7 | |
| Cut-offs based on tertile values | | | | *0.002* |
| ≤ 0.468 | 104/77 | 26.6 | 18.4 – 35.4 | |
| 0.468 – 1.582 | 104/66 | 36.5 | 27.4 – 45.7 | |
| > 1.582 | 106/63 | 40.6 | 31.2 – 49.7 | |
| *Stage 4S* | | | | |
| Cut-off based on the median value | | | | *0.759* |
| ≤ 1.474 | 44/14 | 68.2 | 52.3 – 79.8 | |
| > 1.474 | 46/13 | 71.7 | 56.4 – 82.5 | |
| Cut-offs based on tertile values | | | | *0.672* |
| ≤ 0.882 | 29/9 | 69.9 | 48.8 – 82.5 | |
| 0.882 – 1.800 | 30/10 | 66.7 | 46.9 – 80.5 | |
| > 1.800 | 31/8 | 74.2 | 55.0 – 86.2 | |

N/D = Number of patients/ deaths. OS = Overall Survival, 95%CI: 95% confidence interval of OS. n.e. = not evaluable

**Supplementary Table S4.** Survival of 786 NB patients in relation to *FANCA* expression levels by *MYCN* status.

| Overall Survival | **N/D** | **OS** | **95%CI** | ***p*** |
|---|---|---|---|---|
| *MYCN normal* | | | | |
| Cut-off based on the median value | | | | *< 0.001* |
| ≤ 3.277 | 314/42 | 86.0 | 81.4 – 89.5 | |
| > 3.277 | 315/77 | 75.8 | 70.5 – 80.3 | |
| Cut-offs based on tertile values | | | | *< 0.001* |
| ≤ 2.960 | 209/29 | 85.7 | 80.0 – 89.9 | |
| 2.960 – 3.580 | 210/32 | 84.0 | 78.0 – 88.5 | |
| > 3.580 | 210/58 | 73.0 | 66.2 – 78.6 | |
| *MYCN amplified* | | | | |
| Cut-off based on the median value | | | | *0.927* |
| ≤ 3.945 | 76/53 | 29.6 | 19.6 – 40.3 | |
| > 3.945 | 77/55 | 28.6 | 19.0 – 38.9 | |
| Cut-offs based on tertile values | | | | *0.975* |
| ≤ 3.715 | 51/38 | 24.4 | 13.3 – 37.2 | |
| 3.715 – 4.151 | 51/31 | 39.2 | 26.0 – 52.2 | |
| > 4.151 | 51/39 | 23.5 | 13.0 – 35.8 | |

| Event Free  Survival | **N/E** | **EFS** | **95%CI** | ***p*** |
|---|---|---|---|---|
| Cut-off based on the median value | | | | *< 0.001* |
| ≤ 3.277 | 304/72 | 76.3 | 71.1 – 80.7 | |
| > 3.277 | 310/135 | 56.7 | 51.0 – 62.0 | |
| Cut-offs based on tertile values | | | | *< 0.001* |
| ≤ 2.960 | 202/46 | 77.2 | 70.8 – 82.4 | |
| 2.960 – 3.580 | 205/57 | 72.2 | 65.5 – 77.8 | |
| > 3.580 | 207/104 | 50.2 | 43.2 – 56.8 | |
| *MYCN amplified* | | | | |
| Cut-off based on the median value | | | | *0.793* |
| ≤ 3.945 | 74/54 | 25.8 | 16.0 – 36.7 | |
| > 3.945 | 77/57 | 26.0 | 16.8 – 36.1 | |
| Cut-offs based on tertile values | | | | *0.960* |
| ≤ 3.715 | 49/37 | 24.5 | 13.6 – 37.1 | |
| 3.715 – 4.151 | 51/34 | 31.8 | 19.0 – 45.3 | |
| > 4.151 | 51/40 | 21.6 | 11.6 – 33.6 | |

N/D = Number of patients/ Deaths. OS = Ten-year Overall Survival, N/E Number of patients/ Events. EFS = Ten-year Event Free Survival, 95%CI: 95% confidence interval.

**Supplementary Table S5.** Survival of 786 NB patients in relation to *FANCA* expression levels by INSS stage.

| Overall Survival | N/D | OS | 95%CI | *p* |
|---|---|---|---|---|
| *Localized stage* | | | | |
| Cut-off based on the median value | | | | *< 0.001* |
| ≤ 3.221 | 186/7 | 96.2 | 92.2 – 98.2 | |
| > 3.221 | 187/28 | 85.0 | 79.0 – 89.4 | |
| Cut-offs based on tertile values | | | | *< 0.001* |
| ≤ 2.897 | 124/2 | 98.4 | 93.7 – 99.6 | |
| 2.897 – 3.518 | 124/9 | 92.7 | 86.4 – 96.1 | |
| > 3.518 | 125/24 | 80.7 | 72.6 – 86.6 | |
| *Stage 4* | | | | |
| Cut-off based on the median value | | | | *0.001* |
| ≤ 3.657 | 160/79 | 49.4 | 40.9– 57.3 | |
| > 3.657 | 160/104 | 35.5 | 28.1 – 43.1 | |
| Cut-offs based on tertile values | | | | *< 0.001* |
| ≤ 3.314 | 106/51 | 49.2 | 38.4 – 59.1 | |
| 3.314 – 3.949 | 107/56 | 48.8 | 38.9 – 58.0 | |
| > 3.949 | 107/76 | 29.2 | 20.7 – 38.2 | |
| *Stage 4S* | | | | |
| Cut-off based on the median value | | | | *0.822* |
| ≤ 3.307 | 46/6 | 87.0 | 73.3 – 93.9 | |
| > 3.307 | 46/5 | 87.5 | 71.7 – 94.8 | |
| Cut-offs based on tertile values | | | | *n.e.* |
| ≤ 3.134 | 30/4 | n.e. | n.e. | |
| 3.134 – 3.672 | 31/4 | n.e. | n.e. | |
| > 3.672 | 31/3 | n.e. | n.e. | |

| Event Free Survival | N/E | EFS | 95%CI | *p* |
|---|---|---|---|---|
| *Localized stage* | | | | |
| Cut-off based on the median value | | | | *< 0.001* |
| ≤ 3.221 | 179/22 | 87.7 | 81.9 – 91.7 | |
| > 3.221 | 185/64 | 65.4 | 58.1 – 71.8 | |
| Cut-offs based on tertile values | | | | *< 0.001* |
| ≤ 2.897 | 118/11 | 90.7 | 83.8 – 94.7 | |
| 2.897 – 3.518 | 123/23 | 81.3 | 73.2 – 87.2 | |
| > 3.518 | 123/52 | 57.7 | 48.5 – 65.9 | |
| *Stage 4* | | | | |
| Cut-off based on the median value | | | | *0.003* |
| ≤ 3.657 | 155/90 | 41.9 | 34.1 – 49.6 | |
| > 3.657 | 159/116 | 27.3 | 20.5 – 34.5 | |
| Cut-offs based on tertile values | | | | *< 0.001* |
| ≤ 3.314 | 105/59 | 43.8 | 34.2 – 53.0 | |
| 3.314 – 3.949 | 102/65 | 36.9 | 27.5 – 46.2 | |
| > 3.949 | 107/82 | 23.4 | 15.9 – 31.7 | |
| *Stage 4S* | | | | |
| Cut-off based on the median value | | | | *0.748* |
| ≤ 3.307 | 44/13 | 70.5 | 54.6 – 81.7 | |
| > 3.307 | 46/14 | 69.6 | 54.1 – 80.7 | |
| Cut-offs based on tertile values | | | | *0.221* |
| ≤ 3.134 | 29/7 | 75.9 | 55.9 – 87.7 | |
| 3.134 – 3.672 | 30/9 | 70.0 | 50.3 – 83.1 | |
| > 3.672 | 31/11 | 64.5 | 45.2 – 78.5 | |

N/D = Number of patients/ deaths. OS = Overall Survival, 95%CI: 95% confidence interval of OS. n.e. = not evaluable

**Supplementary Table S6.** Survival of 786 NB patients in relation to *MPAK3* expression levels by *MYCN* status.

| Overall Survival | **N/D** | **OS** | **95%CI** | ***p*** |
|---|---|---|---|---|
| *MYCN normal* | | | | |
| Cut-off based on the median value | | | | *0.195* |
| ≤ 3.880 | 314/53 | 83.5 | 78.9 – 87.3 | |
| > 3.880 | 315/66 | 78.1 | 72.8 – 82.5 | |
| Cut-offs based on tertile values | | | | *0.235* |
| ≤ 3.696 | 209/37 | 82.5 | 76.5 – 87.1 | |
| 3.696 – 4.057 | 210/35 | 83.5 | 77.6 – 87.9 | |
| > 4.057 | 210/47 | 76.6 | 69.7 – 82.0 | |
| *MYCN amplified* | | | | |
| Cut-off based on the median value | | | | *0.194* |
| ≤ 3.625 | 76/51 | 32.9 | 22.7 – 43.5 | |
| > 3.625 | 77/57 | 24.2 | 14.6 – 35.2 | |
| Cut-offs based on tertile values | | | | *0.220* |
| ≤ 3.494 | 51/37 | 27.5 | 16.1 – 40.0 | |
| 3.494 – 3.894 | 51/30 | 41.2 | 27.7 – 54.2 | |
| > 3.894 | 51/41 | 21.6 | 11.6 – 33.6 | |

| Event Free Survival | **N/E** | **EFS** | **95%CI** | ***p*** |
|---|---|---|---|---|
| Cut-off based on the median value | | | | *0.923* |
| ≤ 3.880 | 314/106 | 66.5 | 61.0 – 71.5 | |
| > 3.880 | 300/101 | 66.3 | 60.7 – 71.4 | |
| Cut-offs based on tertile values | | | | *0.940* |
| ≤ 3.696 | 209/74 | 64.6 | 57.7 – 70.6 | |
| 3.696 – 4.057 | 210/63 | 70.5 | 63.8 – 76.2 | |
| > 4.057 | 195/70 | 64.1 | 56.9 – 70.4 | |
| *MYCN amplified* | | | | |
| Cut-off based on the median value | | | | *0.280* |
| ≤ 3.625 | 76/54 | 28.4 | 18.6 – 38.9 | |
| > 3.625 | 75/57 | 24.0 | 15.1 – 34.1 | |
| Cut-offs based on tertile values | | | | *0.480* |
| ≤ 3.494 | 51/38 | 25.5 | 14.6 – 37.9 | |
| 3.494 – 3.894 | 51/34 | 31.8 | 19.0 – 45.3 | |
| > 3.894 | 49/39 | 20.4 | 10.5 – 32.6 | |

N/D = Number of patients/ Deaths. OS = Ten-year Overall Survival, N/E Number of patients/ Events. EFS = Ten-year Event Free Survival, 95%CI: 95% confidence interval.

**Supplementary Table S7.** Survival of 786 NB patients in relation to *MAPK3* expression levels by INSS stage.

| Overall Survival | N/D | OS | 95%CI | *p* |
|---|---|---|---|---|
| *Localized stage* | | | | |
| Cut-off based on the median value | | | | *0.112* |
| ≤ 3.868 | 186/22 | 88.1 | 82.5 – 92.0 | |
| > 3.868 | 187/13 | 93.0 | 88.2 – 95.9 | |
| Cut-offs based on tertile values | | | | *0.027* |
| ≤ 3.652 | 124/20 | 83.9 | 76.1 – 89.3 | |
| 3.652 – 4.038 | 124/5 | 95.9 | 90.4 – 98.3 | |
| > 4.038 | 125/10 | 91.9 | 85.5– 95.6 | |
| *Stage 4* | | | | |
| Cut-off based on the median value | | | | *0.715* |
| ≤ 3.817 | 160/88 | 44.9 | 36.9– 52.6 | |
| > 3.817 | 160/95 | 40.0 | 32.0 – 47.9 | |
| Cut-offs based on tertile values | | | | *0.926* |
| ≤ 3.619 | 106/61 | 42.8 | 33.1 – 52.1 | |
| 3.619 – 4.005 | 107/57 | 47.4 | 37.6 – 56.5 | |
| > 4.005 | 101/65 | 36.7 | 26.7 – 46.8 | |
| *Stage 4S* | | | | |
| Cut-off based on the median value | | | | *0.003* |
| ≤ 3.822 | 46/1 | 97.8 | 85.6 – 99.7 | |
| > 3.822 | 46/10 | 77.8 | 60.6 – 87.0 | |
| Cut-offs based on tertile values | | | | *n.e.* |
| ≤ 3.686 | 30/0 | n.e. | n.e. | |
| 3.686 – 4.057 | 31/7 | n.e. | n.e. | |
| > 4.057 | 31/4 | n.e. | n.e. | |

| Event Free Survival | N/E | EFS | 95%CI | *p* |
|---|---|---|---|---|
| *Localized stage* | | | | |
| Cut-off based on the median value | | | | *0.083* |
| ≤ 3.868 | 186/51 | 72.6 | 65.6 – 78.4 | |
| > 3.868 | 178/35 | 80.3 | 73.7 – 85.5 | |
| Cut-offs based on tertile values | | | | *0.136* |
| ≤ 3.652 | 124/37 | 70.2 | 61.3 – 77.4 | |
| 3.652 – 4.038 | 124/24 | 80.7 | 72.5 – 86.6 | |
| > 4.038 | 116/25 | 78.5 | 69.8 – 84.9 | |
| *Stage 4* | | | | |
| Cut-off based on the median value | | | | *0.372* |
| ≤ 3.817 | 160/108 | 32.8 | 25.6 – 40.2 | |
| > 3.817 | 154/98 | 36.4 | 28.8 – 43.9 | |
| Cut-offs based on tertile values | | | | *0.469* |
| ≤ 3.619 | 106/71 | 32.4 | 23.5 – 41.5 | |
| 3.619 – 4.005 | 107/70 | 35.5 | 26.6 – 44.5 | |
| > 4.005 | 101/65 | 35.6 | 26.5 – 44.9 | |
| *Stage 4S* | | | | |
| Cut-off based on the median value | | | | *0.032* |
| ≤ 3.822 | 46/9 | 80.4 | 65.8 – 89.3 | |
| > 3.822 | 44/18 | 59.1 | 43.2 – 71.9 | |
| Cut-offs based on tertile values | | | | *0.101* |
| ≤ 3.686 | 30/5 | 83.3 | 64.5 – 92.7 | |
| 3.686 – 4.057 | 31/11 | 64.5 | 45.2 – 78.5 | |
| > 4.057 | 29/11 | 62.1 | 42.1 – 76.9 | |

N/D = Number of patients/ deaths. OS = Overall Survival, 95%CI: 95% confidence interval of OS. n.e. = not evaluable

**Supplementary Table S8.** Main characteristics of the 786 NB patients selected for the analyses.

| Patient characteristics | N | % |
|---|---|---|
| *Age at diagnosis* | | |
| < 18 months | 449 | 57.1 |
| ≥ 18 months | 337 | 42.9 |
| *Disease extension* | | |
| Localized | 373 | 47.5 |
| Disseminated | 412 | 52.4 |
| Missing | 1 | 0.1 |
| *INSS stage* | | |
| 1 | 143 | 18.2 |
| 2 | 125 | 15.9 |
| 3 | 105 | 13.4 |
| 4 | 320 | 40.7 |
| 4S | 92 | 11.7 |
| Missing | 1 | 0.1 |
| *MYCN status* | | |
| Not amplified | 629 | 80.0 |
| Amplified | 153 | 19.5 |
| Missing | 4 | 0.5 |
| Events [1] | 320 | 40.7 |
| Deaths | 229 | 29.1 |

[1] 17 missing for the Event Free Survival.
